# A Prospective Observational Study on Multiplate^®^-, ROTEM^®^- and Thrombin Generation Examinations Before and Early After Implantation of a Left Ventricular Assist Device (LVAD)

**DOI:** 10.3389/fmed.2022.760816

**Published:** 2022-02-25

**Authors:** Philipp Opfermann, Alessia Felli, Christine Schlömmer, Martin Dworschak, Michele Bevilacqua, Mohamed Mouhieddine, Daniel Zimpfer, Andreas Zuckermann, Barbara Steinlechner

**Affiliations:** ^1^Department of Anesthesia, Intensive Care Medicine and Pain Medicine, Division of General Anesthesia and Intensive Care, Medical University of Vienna, Vienna, Austria; ^2^Department of Anesthesia, Intensive Care Medicine and Pain Medicine, Division of Cardiothoracic and Vascular Anesthesia and Intensive Care, Medical University of Vienna, Vienna, Austria; ^3^Department of Cardiac Surgery, Medical University of Vienna, Vienna, Austria

**Keywords:** bleeding, left ventricular assist device (LVAD), point of care coagulation tests, rotational thromboelastometry (ROTEM), platelet aggregometry, thrombin generation

## Abstract

**Background:**

Heart failure patients are frequently on coagulation-active medications before LVAD implantation and perioperative bleeding is a frequent complication after left ventricular assist device (LVAD) implantation. The role of point-of-care coagulation tests in assessing bleeding risk for LVAD implantation and the early postoperative time course of these tests is not well established.

**Methods:**

We prospectively enrolled 25 patients with terminal heart failure undergoing LVAD implantation. Study related TRAP-, ASPI- and ADP- tests of Multiplate^®^ platelet aggregometry, ROTEM^®^ rotational thromboelastometry (INTEM, EXTEM, FIBTEM), thrombin generation assay and conventional laboratory studies were measured at 11 predefined time-points during the first 21 postoperative days. We examined if preoperative TRAP-, ASPI-, ADP- and ROTEM values are correlated with estimated total blood loss (primary outcome parameter) during the first 21 days after LVAD implantation and compared the baseline values of these measurements between patients with a bleeding event to those without. We performed Spearman's correlation and non-parametric tests for paired and non-paired comparisons.

**Results:**

7 out of 25 (28%) patients experienced a bleeding event of which 4 required surgical revision. Of the preoperatively performed measurements the TRAP test [Spearman's Rho (ρ) = −0.5, *p* = 0.01], INTEM CFT (ρ = 0.72, *p* < 0.001), INTEM alpha (−0.7, *p* < 0.001), EXTEM MCF (ρ = −0.63; *p* < 0.001), EXTEM alpha (ρ = −0.67; *p* < 0.001), FIBTEM MCF (ρ = −0.41; *p* = 0.042), Fibrinogen (Clauss) (ρ = −0.5; *p* = 0.011), Anti-thrombin activity (ρ = −0.49; *p* = 0.013) and platelet count (ρ = −0.42; *p* = 0.034) were significantly correlated to total blood loss. Patients undergoing a surgical bleeding revision had significantly reduced values in TRAP—[31.5 IQR (17.25–43.5U) vs. 69 IQR (52.5–87U); *p* = 0.004], ASPI—[16.5 IQR (5.5–35.7U) vs. 39 IQR (24.5–62.5U); *p* = 0.038], ADP—[30 IQR (22–69U) vs. 12.5 IQR (8.7–21.5U); *p* = 0.01], EXTEM MCF—[63 IQR (57.7–63.7) vs. 67 IQR (65–75.5); *p* = 0.019] and EXTEM alpha [74 IQR (68.75–74) vs. 79 IQR (78–80.5); *p* = 0.002] values before LVAD implantation.

**Conclusion:**

Multiplate^®^ and ROTEM^®^ measurements before LVAD implantation may identify LVAD candidates with platelet dysfunction and alterations of the primary hemostasis and could guide anesthetists and intensive care practitioners in bleeding risk stratification and in the perioperative clinical management.

## Introduction

Implantation of a left ventricular assist device (LVAD) can be life—saving for patients with end-stage heart failure (1). Accordingly, indications for implantation have broadened in recent years (2). However, bleeding, thromboembolism, and infections after surgery still cause considerable morbidity and mortality in this patient population (1, 3–5). Early bleeding is a common complication after LVAD implantation, requiring transfusion of blood products resulting in increased all-cause mortality (6) and acute RV failure (7). LVAD candidates are often on anticoagulants and antiplatelet drugs before LVAD implantation according to the underlying etiology of terminal heart failure. Particularly for the anesthetist, the intensive care practitioner and the surgeon the sustained impact of anticoagulant and antiplatelet drugs can be a major challenge in the early clinical management for LVAD implantation and for the first weeks after device implantation. Furthermore, platelet function monitoring have been suggested to assist decision-making about the timing of surgery after cessation of antiplatelet drugs, minimizing unnecessary delay, and reducing exposure of the patient to an increased risk of bleeding complications (8).

At our center standard hemostatic laboratory tests performed prior to LVAD implantation [e.g., prothrombin time, activated partial thromboplastin time, fibrinogen (Clauss method), platelet count] (9, 10) are part of a routine preoperative evaluation of the patient. However, this standard hemostatic panel used in daily routine does neither reflect a preexisting platelet dysfunction or the degree of platelet inhibition, nor is it a good measure for primary hemostasis. It has been shown that point-of-care (POC) testing to assess coagulation and platelet activation before major surgery can reduce the need for transfusion (11, 12) as well as morbidity and mortality (13, 14). Additionally, ROTEM^®^-guided bleeding management has been shown to be superior to conventional management of bleeding in patients undergoing complex cardiac surgery in terms of reduced postoperative blood loss (15). Furthermore, POC allows the anesthetists to make rapid clinical decisions (9) about patient care in the OR during LVAD implantation and has been recommended in the European guidelines for the treatment of massive postoperative bleeding to achieve a timely hemostatic intervention (16). Two of these point-of-care devices available at our center are the Rotation thromboelastometry (ROTEM^®^Delta, TEM Innovations GmbH, Munich, Germany), which is a modification of the classical thromboelastography and the whole-blood impedance aggregometry (Multiplate^®^, Roche Germany Holding GmbH). In contrast to classical coagulation measurements that are performed on cell-free plasma samples, ROTEM^®^ is performed in whole-blood samples (17). ROTEM^®^ provides information on the contribution of fibrinogen and platelets to clot formation and measures the viscoelastic properties of a blood clot as it forms *in vitro* (17). However, the method is insensitive to antithrombin, protein C, protein S or thrombomodulin. The Multiplate^®^ device allows platelet function to be assessed by using anticoagulated whole blood as milieu without any sample processing (18). It measures the platelets' ability to form aggregates in response to different agonists (e.g., TRAP, ASPI, ADP) (19). Thrombin is one of the most potent agonists in this context signaling through the protease-activated receptor (PAR)-1 receptor (20–22). The current literature does not provide literature on pre- and early post-operative use of ROTEM^®^ and Multiplate^®^ in heart failure patients undergoing LVAD implantation. Therefore, the aim of this prospective non-interventional pilot study was to evaluate if values in Multiplate^®^- (TRAP test, ASPI test and ADP test) and ROTEM^®^- (INTEM, EXTEM and FIBTEM) examinations measured before LVAD implantation are significantly correlated to enhanced estimated total blood loss during and after LVAD implantation. A further aim was to compare the baseline coagulation measurements before LVAD implantation of patients with subsequent bleeding event (s) during the first 21 days after LVAD implantation to those without bleeding events. We hypothesize that reduced Multiplate^®^- and ROTEM^®^ alpha angle/MFC values are correlated to enhanced blood loss and a higher probability of significant bleeding. Furthermore we describe the temporal pattern of point-of-care parameters and thrombin-generation measurements during the first 21 days after LVAD implantation.

## Materials and Methods

This observational study, employing a prospective longitudinal design, was performed at the General Hospital Vienna, a tertiary care center. The ethical review committee of the Medical University of Vienna granted approval for this study (EK-Nr: 1625/2013).

### Study Population

All patients with terminal heart failure irrespective of etiology, ≥18 years of age scheduled for LVAD implantation by the cardiac surgeons (either as “bridge to transplant”, “bridge to candidacy”, “bridge to recovery”, or “destination therapy”) and > INTERMACS 1 were eligible for inclusion in this study. The ethics committee gave approval only for inclusion of patients who could consent before LVAD implantation. Therefore exclusion criteria were children and adolescents, INTERMACS 1 patients supported by ECMO and/or intubated before LVAD implantation and patients who were not able to consent due to sedative drugs or critical illness. After formal information of our study team by the attending surgeons that a LVAD candidate has been admitted, a primary survey of the medical records by our study team has been done to check for eligibility. After an informal patient interview the patient signed the informed consent form and a further evaluation of the medical records have been done. By patient history and studying the medical records the study baseline characteristics (e.g., age, sex, INTERMACS grade, comorbidities and medications, echocardiography findings) have been determined.

Blood collection was done at the following time points: Before LVAD implantation (= baseline values “as admitted for LVAD implantation”), immediately after admission to the ICU following LVAD implantation, daily between postoperative days (POD) 1-7, and again on POD 14 and 21 at 9.00 a.m. Blood was drawn for Multiplate^®^, ROTEM^®^ and Thrombin generation examinations. The patient's clinical course based on inpatient and outpatient records was followed until the end of the first postoperative year.

### LVAD Implantation

The choice of a Heartware^®^ (HVAD) or Thoratec^®^ Heartmate (HM II or III) device implantation as well as the implantation strategy was left at the discretion of the attending surgeon and was performed according to surgical standard operation procedure (SOP) following international recommendations. Whenever possible our teams of surgeons apply a minimally invasive approach of LVAD implantation via bilateral minithoracotomy in HVAD or subcostal incision and right mini-thoracotomy in HM II as described elsewhere in detail (6, 23). A full sternotomy approach was reserved for post-cardiotomy patients and patients with a history of previous thoracotomy.

### Clinical Outcomes

The primary outcomes of the study were total volume of blood loss and the occurrence of major or minor bleeding during the first 3 weeks after LVAD implantation. We calculated total blood loss as the sum of intraoperative (including any surgical revision) estimated blood loss (ml) and the output from indwelling chest drains (ml) till their removal. A study team member not being involved in the treatment process of the patient estimated the intraoperative blood loss. Blood that could not have been re-transfused after intraoperative blood salvage, blood in the detritus suction, blood of the extracorporeal circuit not utilized for re-transfusion and surgical dressing or wipes used intra-operatively have been considered. At the end of the standard surgical treatment, two or three chest tubes were placed in the mediastinal and/ or pleural space to continuously monitor postoperative blood loss and to prevent undesirable blood collection. After transfer to the ICU the output from the indwelling chest tubes were measured by the attending medical team. This was done every hour during the first 24 h after ICU admission and then every 4 h. The indwelling chest tubes were removed at discretion of the attending intensive care practitioner and the surgeon when the patient was stable enough to do so, no bleeding from the surgical side was apparent and the output was predominantly serous fluid.

Major bleeding was defined according to institutional guidelines (6) as hemorrhagic drainage exceeding 200 mL/h with a concomitant drop in hemoglobin during the first postoperative hours, new onset of significant bleeding with hemodynamic instability or cardiopulmonary resuscitation in the presence of mediastinal hematoma documented by echocardiography or computed tomography that required surgical revision. In this cases reoperative surgery was performed whenever a source of bleeding was suspected to be related to surgical site and optimization of coagulation according to institutional bleeding guidelines by the ICU staff did not improve the bleeding situation. Minor bleeding was defined as confirmed gastrointestinal bleeding or bleeding from other locations (e.g., bleeding from nose), which did not require surgical intervention. Major and minor bleeding patients were simplistically summarized as “bleeders” and compared to “non-bleeders” because of the small number of patients.

Secondary outcomes were the total amount of blood products [packed red blood cells (PRBCs), fresh frozen plasma (FFP) or platelet concentrates] and the frequency of thromboembolic events during the same period, as well as all-cause death until the end of the first postoperative year. All patients were followed for the consumption of PRBCs, FFP or platelet concentrates for the first 3 weeks after LVAD implantation.

All professional involved in the clinical treatment process did not have insight any of our study related measurements. We underline that this examination is a non-interventional observation study of a routine clinical management pathway. This means explicit that no treatment decision was done based on study related coagulation measurements.

The transfusion of PRBCs, PLT and FFPs followed the 2017 EACTS/EACTA Guidelines on patient blood management for adult cardiac surgery (24) and the Guidelines from the European Society of Anaesthesiology for the management of severe perioperative bleeding (16). Those guidelines are implemented in the SOP at our center.

### Platelet Aggregometry (Multiplate^®^ Analyzer)

Platelet function was assessed using the Multiplate^®^ Analyzer (Roche Pharma AG, Grenzach-Wyhlen, Germany) (25). Blood was drawn into vials containing hirudin as anticoagulant, mixed with the same volume of 0.9% saline solution, and incubated for 3 min. After stirring at 37°C, the platelet agonists arachidonic acid, thrombin receptor activator peptide-6 (TRAP-6; 32 μM) and ADP were added, and aggregation was continuously recorded over 5 min. Aggregation was quantified as the area under the curve, an integrated measure of velocity and maximal aggregation expressed in units (U).

### Rotational Thromboelastometry (ROTEM^®^ Delta System)

To evaluate the role of coagulation factors, their inhibitors, and cellular components in hemostasis, we measured the changes in elasticity at all stages of the developing and resolving clot by ROTEM (ROTEM^®^ delta system, TEM Innovations GmbH, Munich, Germany) analysis. This system allows the differential diagnosis of multifactorial coagulopathy by testing extrinsic (EXTEM test: hemostasis activated by tissue factor) and intrinsic (INTEM test: hemostasis activated by ellagic acid) coagulation pathways, as well as the measurement of fibrin polymerization (FIBTEM test: platelet inhibition by cytochalasin D isolates fibrinogen function). We employed three separate assays activated by either ellagic acid (INTEM reagent), tissue factor (EXTEM reagent), or tissue factor and the platelet inhibitor cytochalasin D (FIBTEM reagent).

### Measurement of Thrombin Generation

Thrombin generation was assessed using the Thrombinoscope^®^ (Stago Austria GmbH, Vienna) (26). In brief, the device monitors the fluorescence generated by thrombin cleavage of a fluorogenic substrate over time upon activation of the coagulation cascade by different concentrations of tissue factor and negatively charged phospholipids in plasma. From the temporal changes in fluorescence, the concentration of thrombin can be calculated and the rate of increase in thrombin concentration over time then allows calculation of thrombin generation. The following parameters are derived from the thrombogram: lag time (min), endogenous thrombin potential (ETP; nM^*^min), peak thrombin (nM), time to peak (min), velocity index (nM/min), and start tail (min) (27). Coagulation was activated using the Technothrombin^®^ TGA reagent C (RC) containing a high concentration of phospholipid micelles with rhTF in Tris-Hepes-NaCl buffer. Blood was collected into 0.1 mL vials containing 3.6% trisodium citrate. Samples were centrifuged for 20 min at 2,000 × g to get Platelet poor plasma and stored at −80°C until final analysis. To show the course of thrombin generation over time for each individual, lag time, maximal rate of thrombin generation, peak thrombin, and endogenous thrombin potential (total thrombin) were plotted against time using a Motion Chart (28) that generates a five-dimensional plot. In these plots, lag time is shown on the y-axis and maximal rate of thrombin on the x-axis. Peak thrombin is represented by the color, and endogenous thrombin potential by the relative size of each data point. The time component is shown by animating each point to move as thrombin generation parameters change over time.

### Anticoagulation Regimen

Anticoagulation was managed according to our institutional guidelines (29) using either unfractionated or low-molecular-weight heparin (LMWH). LMWH was usually started within the first 24 h postoperatively but could be delayed due to prolonged postoperative bleeding. Enoxaparin was given at an initial dose of 0.5 mg/kg rounded to administer available dosages of 40, 60, or 80 mg targeting a peak anti-Xa activity of 0.2 to 0.4 IU/mL. Alternatively, unfractionated heparin was given to reach and maintain an activated partial thromboplastin time (aPTT) of 50–55 s. Oral anti-coagulation with phenprocoumon was started after removal of chest tubes and indwelling catheters when no further interventions were to be expected and oral intake of medication was satisfactory. LMWH or unfractionated heparin was continued until a target international normalized ratio (INR) of 2–2.5 was achieved. Anti-platelet therapy was started on the third postoperative day and consisted of 100 mg aspirin daily in HM II or 100 mg aspirin twice daily in HVAD patients.

All attending anesthesiologists, surgeons, physicians and persons who were involved in the clinical treatment of the patients were blinded to the results of the study related Multiplate^®^, thromboelastometry, and thrombin-generation measurements. No treatment decision was carried out based on these findings.

### Data Collection

All data collected per patient was initially collected in hard copy using a pre-specified case report form. Sources of data were the interview and patient history, the medical records at admission, the hospital real time databases AKH-PDMS (Philips Healthcare, Vienna, Austria) and AKIM (AKH Information Management, Vienna, Austria) and the discharge records. A member of the study team was present in the OR and at ICU admission of the patient and did a least a daily evaluation of the clinical status of the patient and for completion of the case report form from the first postoperative day onwards. The collected data was transferred further to a SPSS database after screening for completeness, consistency and outliers.

### Statistics

Patient characteristics are described using conventional summary statistics, i.e., medians with interquartile ranges (IQRs) or absolute numbers (percentages). Continuous variables were compared using the Mann-Whitney U-test. For paired data the non-parametric Wilcoxon test was used. Proportions were analyzed using Fisher's exact or Pearson's Chi Square test.

We calculated total blood loss as the sum of intraoperative blood loss and the output from chest drains placed at the end of the surgical standard care. This calculated sum was used as the primary endpoint and for the Spearman's correlation analysis. Spearman's correlation coefficient (r) was calculated to measure the correlation between two sets of data. All tests were two-sided. Differences were considered significant when *p* < 0.05. Violin plots were used for visualization of the comparison of metric variables between two groups showing the median with IQR and the density plot width. SPSS^®^ Statistics (Version 24.0.0.0, IBM, Armonk, NY) and GraphPad Prism^®^ (Version 8.0.2, GraphPad Software, San Diego, CA) were used for statistical analyses.

## Results

### Patient Characteristics and Clinical Outcomes

Twenty-five patients undergoing LVAD implantation were included in this study. The etiology of heart failure was ischemic in 17 and dilated cardiomyopathy in 8 patients. Detailed baseline characteristics of patients are given in Table 1. Ten patients received a HeartWare^®^ (HVAD) and 15 a HeartMate^®^ (HMII or III) device (Table 2). Bleeding occurred in 7 out of the 25 patients within the 21-day observation period. During this time, four patients had a major bleeding requiring surgical revision, two of them because of hemothorax, one due to pericardial effusion, and one because of bleeding as a result of right ventricular injury. Three patients developed a gastrointestinal bleeding episode and another three patients had thromboembolic complications (including one pump thrombosis) during the first 3 weeks after LVAD implantation. Table 3 summarizes the consumption of blood products during the observation period compared between bleeders and non-bleeders. However, median follow-up time was 460 days (IQR 229–585 days). The 30, 90-day, and 1-year mortality rates were 0, 16, and 28%, respectively. Causes of death at 1 year are summarized in Supplementary Table 1.

**Table 1:** Preoperative characteristics.

	**Bleeders ^#^(*n =* 7)**	**Non-bleeders (*n =* 18)**	***P-value***
**Age (y)**	67 (66–72)	60 (56–66)	0.025^*^
**Weight (kg)**	80 (76–95)	78 (67–87)	0.38^*^
**Female sex**	1 (14.3)	6 (33.3)	0.62^+^
**Ntpro-BNP (pg/mL)**	5,492 (2,297–6,818)	5,679 (2,621–7,010)	0.74^*^
**INTERMACS level**			0.92^+^
II	2 (28.6)	4 (22.2)	
III	4 (57.1)	10 (55.6)	
IV	1 (14.3)	4 (22.2)	
**Intent**			0.33^+^
Bridge to decision	0 (0)	1 (5.6)	
Bridge to candidacy	3 (42.9)	11 (61.1)	
Bridge to transplantation	0 (0)	2 (11.1)	
Destination therapy	4 (57.1)	4 (22.2)	
**Diabetes**			0.054^+^
NIDDM	2 (28.6)	0 (0)	
IDDM	2 (28.6)	5 (27.8)	
**History of arterial hypertension**	5 (71.4)	10 (55.6)	0.65^+^
**Atrial fibrillation**	5 (71.4)	9 (50)	0.4^+^
**CHA**_**2**_ **DS**_**2**_ **-VASc score**	3 (3–5)	3.5 (2–4)	0.29^*^
**COLD**	2 (28.6)	6 (33.3)	1^+^
**Renal Insufficiency**			0.039^+^
acute	1 (14.3)	1 (5.6)	
chonic	6 (85.7)	7 (38.9)	
**Left ventricular ejection fraction (%)**	21 (10–28)	20 (15–20)	0.42^*^
**Systolic PAP (mmHg)**	55 (38–67)	57 (43–68)	0.74^*^
**Types of cardiomyopathy (CMP)**			1
Ischemic CMP	5 (71.4)	12 (66.7)	
Dilated CMP	2 (28.6)	6 (33.3)	
**Fibrinogen (mg/dL)**	372 (326–482)	408 (351–571)	0.45^*^
**Prothrombin ratio (%)**	58 (49–76)	61 (40–83)	0.74^*^
**aPTT (sec.)**	41 (39–46)	36 (34–41)	0.025^*^
**Platelet count (x 10** ^**9**^ **/L)**	183 (153–221)	238 (189–287)	0.1^*^
**Antiplatelet therapy (before LVAD implantation)**			
Acetylsalicyl acid (100mg/d)	2 (28.6)	7 (38.9)	1^+^
Clopidorel (75 mg/d)	2 (28.6)	2 (11.1)	0.54^+^
Prasugrel (5 mg/d)	0 (0)	1 (5.6)	1^+^
**LMWH (before LVAD implantation)**	2 (28.6)	13 (72.2)	0.075^+^
**Phenprocoumon (before LVAD implantation)**	3 (42.9)	5 (27.8)	0.64^+^

# *Summarizes all patients, with major or minor bleeding; Values are medians with interquartile ranges (IQRs) or absolute numbers (percentages)*;

* *Mann-Whitney-U test*;

+ *Fisher's Exact- or Chi-Square- test; BMI, body mass index; BNP, brain natriuretic peptide; INTERMACS, interagency registry for mechanically assisted circulatory support; NIDDM, non–insulin-dependent diabetes mellitus; CHA2DS2-VASc score, Congestive heart failure, Hypertension, Age > 75, Diabetes, prior Stroke/transient ischemic attack; IDDM, insulin-dependent diabetes mellitus; COLD, chronic obstructive lung disease; HF, hemofiltration; PAP, pulmonary arterial pressure; CMP, cardiomyopathy; aPTT, activated partial thromboplastin time; LMWH, low molecular weight heparin*.

**Table 2:** Intraoperative Data (referring to LVAD implantation).

	**Bleeders^#^**	**Non-bleeders**	***P*-value**
	**(*n =* 7)**	**(*n =* 18)**	
**Operation time (minutes)**	327 (255–370)	260 (179–314)	0.085^*^
**Surgical access**			1^+^
Sternotomy	4 (57.1)	9 (50)	
Minimally invasive	3 (42.9)	9 (50)	
**Circulatory support**			0.39^+^
ECMO	1 (14.3)	2 (11.1)	
HLM	6 (85.7)	12 (66.7)	
Off-pump	0 (0)	4 (22.2)	
**Type of device**			0.18^+^
HVAD	1 (14.3)	9 (50)	
HM (II+III)	6 (85.7)	9 (50)	

# *summarizes all patients, with major or minor bleeding; Values are medians with interquartile ranges (IQRs) or absolute numbers (percentages)*;

* *Mann-Whitney-U test*;

+ *Fisher's Exact- or Chi-Square- test; LVAD, left ventircular assist device; ECMO, extracorporeal membrane oxygenation; HLM, heart-lung-machine; HVAD, HeartWare^®^ HM, HeartMate^®^*.

**Table 3:** Transfused blood products within observation period.

	**Bleeders ^#^**	**Non-bleeders**	***P-value***
	**(*n =* 7)**	**(*n =* 18)**	
Transfused PRBC units	14 (10–21)	4 (1.75–5.25)	0.001^*^
Transfused FFP units	7 (0–15)	0 (0–3)	0.025^*^
Transfused platelet units	3 (2–4)	1 (0–2)	0.017^*^
Fibrinogen (grams)	4 (2–7)	2 (0–4)	0.047^*^
Cryoprecipitates (Beriplex^®^ P/N CSL Behring) in I.E. ^§^	2,000 (0–4,000)	1,500 (750–2,000)	0.65^*^

# *Summarizes all patients, with major or minor bleeding; Values are medians with interquartile ranges (IQRs)*;

* *Mann-Whitney-U test; PRBC, packed red blood cells; FFP, fresh frozen plasma*;

§ *Containing coagulation factors II, VII, IX, X, protein C and S*.

### Results of Mutiplate^®^ Impedance Aggregometry

**Figure 1:**
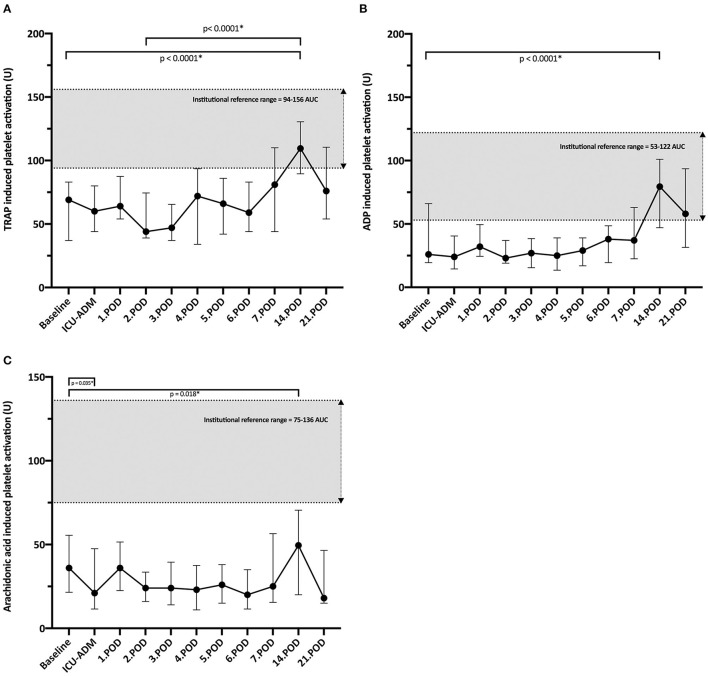
**(A)** Time course of TRAP-induced platelet aggregation (TRAP, thrombin receptor activator peptide-6) measured by the Multiplate^®^ Analyzer (Roche Pharma AG, Grenzach-Wyhlen, Germany); U, units; POD, postoperative day; *Non-parametric Wilcoxon test for paired samples. **(B)** Time course of ADP- induced platelet aggregation; ADP- induced platelet aggregation by the Multiplate^®^ Analyzer (Roche Pharma AG, Grenzach-Wyhlen, Germany); U, units; POD, postoperative day; *Non-parametric Wilcoxon test for paired samples. **(C)** Time course of Arachidonic acid -induced platelet aggregation. Arachidonic acid -induced platelet aggregation by the Multiplate^®^ Analyzer (Roche Pharma AG, Grenzach-Wyhlen, Germany); U, units; POD, postoperative day; *Non-parametric Wilcoxon test for paired samples.

**Figure 2:**
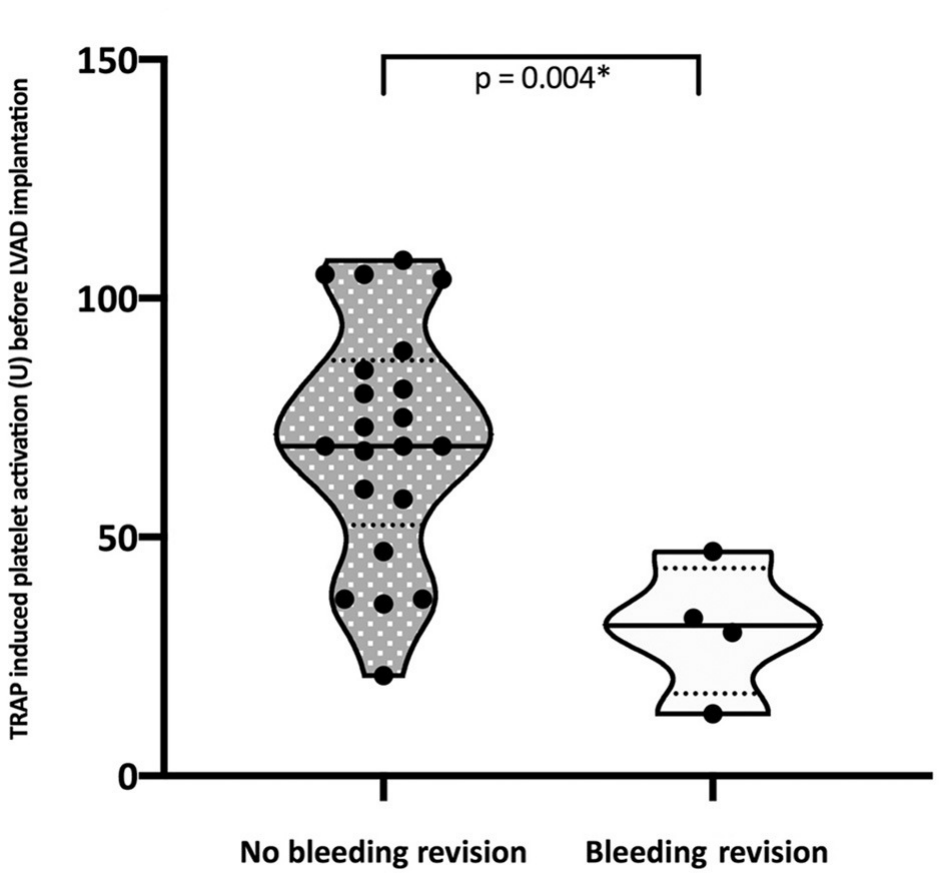
TRAP-induced platelet aggregation before LVAD implantation in patients with and without surgical bleeding revision (TRAP, thrombin receptor activator peptide-6) measured by the Multiplate^®^ Analyzer (Roche Pharma AG, Grenzach-Wyhlen, Germany); the violin plots showing median with IQR and the density plot width; U, units; *Non-parametric Mann-Whitney U-test.

We observed a high frequency of reduced TRAP-induced platelet response below the lower institutional cut-off value of 94 U before LVAD implantation (*n* = 21; 84%). Median TRAP-induced platelet activation was even more reduced on POD2 before recovering to reference values by POD 14 (*p* < 0.0001; Figure 1A). The four patients who underwent surgical bleeding revision after LVAD implantation showed a significantly lower platelet response before LVAD implantation compared to those patients without bleeding revision (Figure 2). Interestingly, those patients who received a HM (II and III) had a slightly lower median baseline TRAP test values, although differences did not reach significance (47; IQR: 30–75 U vs. 48; IQR: 33.75–93.75 U vs. 76.5; IQR: 68.75–86; *p* = 0.159). Results of Spearman's correlation analysis are given in Table 4.

**Table 4:** Spearman's correlation analysis for the relationship of *preoperatively determined* coagulation (= before LVAD implantation) parameters with the estimated total blood loss (ml) during observation period.

**Parameters determined before LVAD implantation**	**Spearman's Rho (95%CI)**	***p*—value^#^**
**Platelet function by Multiplate** ^®^		
TRAP test (U)	−0.51 (−0.76 to −0.1)	0.01
ASPI test (U)	−0.38 (−0.68 to 0.02)	0.056
ADP test (U)	−0.30 (−0.63 to 0.11)	0.14
**ROTEM** ^®^		
EXTEM CT (sec)	−0.31 (−0.43 to −0.37)	0.88
EXTEM CFT (sec)	0.1 (−0.31 to 0.48)	0.61
EXTEM alpha (°)	−0.671 (−0.84 to −0.36)	<0.0001
FIBTEM MCF (mm)	−0.41 (−0.69 to −0.005)	0.042
INTEM CT (sec)	0.29 (−0.12 to 0.62)	0.15
INTEM CFT (sec)	0.722 (0.45 to 0.87)	<0.0001
INTEM alpha (°)	−0.701 (−0.86 to −0.41)	<0.0001
**Standard coagulation parameters**		
Activated partial thromboplastin time (sec)	0.3 (−0.11 to 0.63)	0.13
Antithrombin activity (%)	−0.49 (−0.74 to −0.1)	0.013
Fibrinogen (mg)	−0.501 (−0.75 to −0.11)	0.011
Platelet count (10^9^/L)	−0.426 (−0.7 to −0.02)	0.034
Prothrombin ratio (%)	−0.223 (−0.57 to 0.20)	0.284
**Thrombin generation parameters**		
Endogenous thrombin potential (nM*min)	0.046 (−0.36 to 0.44)	0.827
Lag time (min)	−0.217 (−0.57 to 0.2)	0.297
Peak thombin (nM)	0.072 (−0.34 to 0.46)	0.73
Velocity index (nM/min)	0.069 (−0.34 to 0.46)	0.74

# *Spearman's correlation; LVAD, left ventricular assist device; U, units; sec, seconds; nM, nanomol; min, minutes*.

Arachidonic acid-induced platelet aggregation (ASPI test) declined significantly from baseline (*p* = 0.035) to its nadir at ICU admission. Median ASPI aggregation remained below 50 U during the first postoperative week, and although it showed a significant increase by POD 14, values remained below the lower cut-off value of 75 U during the first 3 postoperative weeks (Figure 1C). However, arachidonic acid-induced platelet values before LVAD implantation did not correlate with total blood loss (Table 4). Patients on Aspirin^®^ (100 mg/ day) medication before LVAD implantation had significantly reduced baseline ASPI test values [23 IQR (11–32 U) vs. 46 IQR (27-67 U); *p* = 0.007] and also patients who underwent surgical bleeding revision after LVAD implantation {ASPI-Test [16.5 IQR (5.5-35.7 U) vs. 39 IQR (24.5-62.5 U); *p* = 0.038]}.

Surprisingly, ADP-induced platelet activation showed a relatively constant time course below the reference range till the 7th POD and showed a significant increase by the 14th (Figure 1B). Noteworthy, only two of our patients were on ADP antagonist before LVAD implantation. ADP-test values before LVAD implantation did not correlate with total blood loss (Table 4). However, similarly to ASPI-measurements at baseline, the ADP-values before LVAD implantation were significantly reduced in patients who required surgical bleeding revision after LVAD implantation [30 IQR (22–69 U) vs. 12.5 IQR (8.7–21.5 U); *p* = 0.01].

We did not observe any relationship between preoperative values of TRAP-, ADP- or arachidonic acid-induced platelet response and thromboembolic complications.

### Results of ROTEM^®^ Analysis

**Figure 3:**
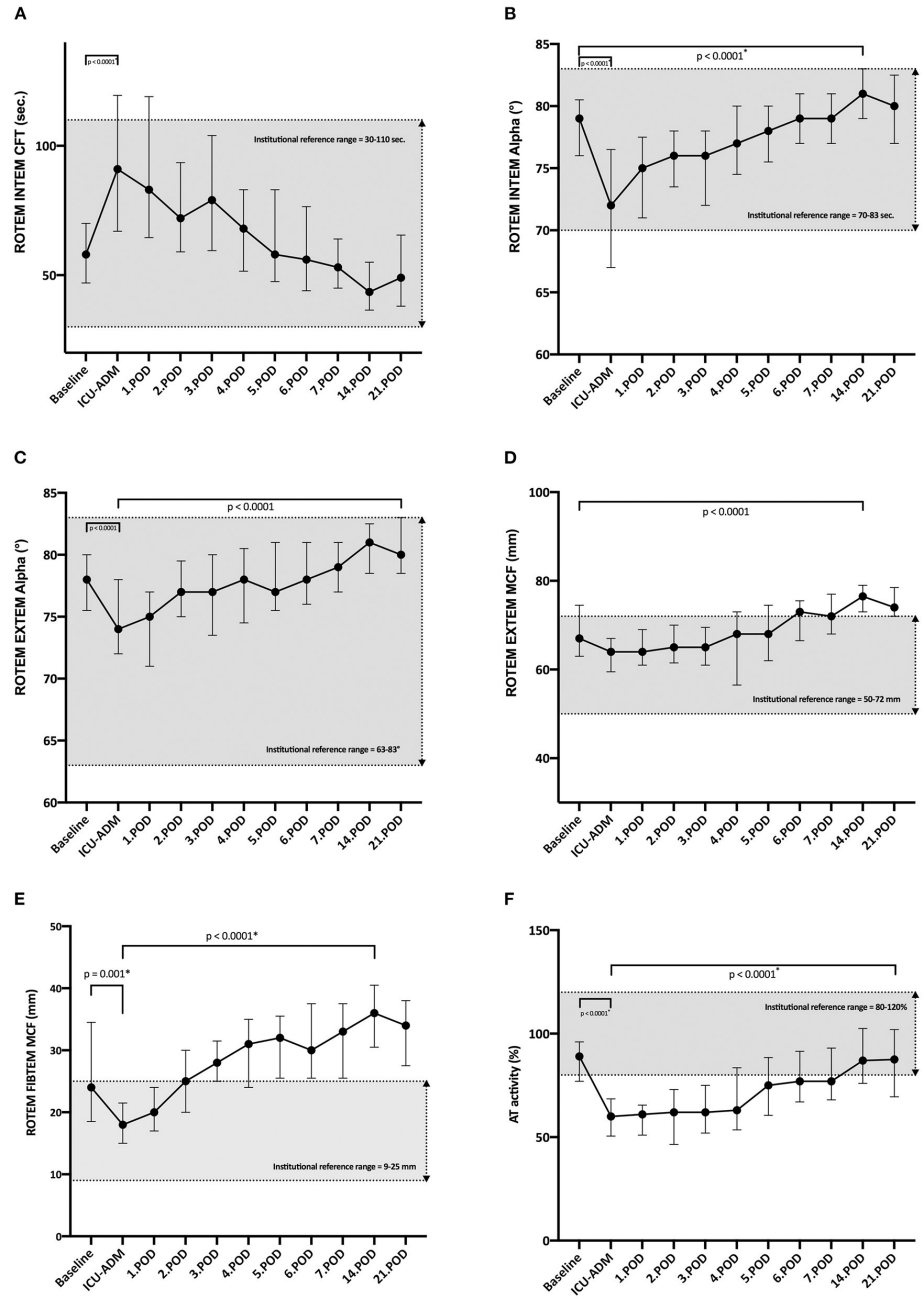
**(A)** Time course of ROTEM^®^ INTEM CFT. Whole blood coagulation profiles determined by rotational thromboelastometry (TEM Innovations GmbH; Munich, Germany) with ellagic acid INTEM reagent (TEM Innovations GmbH); sec, seconds; POD, postoperative day; *Non-parametric Wilcoxon test for paired samples. **(B)** Time course of ROTEM^®^ INTEM alpha. Whole blood coagulation profiles determined by rotational thromboelastometry (TEM Innovations GmbH; Munich, Germany) with ellagic acid INEM reagent (TEM Innovations GmbH); °, Alpha angle; POD, postoperative day; *Non-parametric Wilcoxon test for paired samples. **(C)** Time course of ROTEM^®^ EXTEM alpha. Whole blood coagulation profiles determined by rotational thromboelastometry (TEM Innovations GmbH; Munich, Germany) with tissue factor EXTEM reagent (TEM Innovations GmbH); °, Alpha angle; POD, postoperative day; *Non-parametric Wilcoxon test for paired samples. **(D)** Time course of ROTEM^®^ EXTEM MCF. Whole blood coagulation profiles determined by rotational thromboelastometry (TEM Innovations GmbH; Munich, Germany) with tissue factor EXTEM reagent (TEM Innovations GmbH); MCF, Maximum clot firmness in mm; POD, postoperative day; *Non-parametric Wilcoxon test for paired samples. **(E)** Time course of ROTEM FIBTEM MCF. Whole blood coagulation profiles determined by rotational thromboelastometry (TEM Innovations GmbH; Munich, Germany) with the platelet inhibitor cytochalasin D contained in FIBTEM reagent (TEM Innovations GmbH); MCF, Maximum clot firmness in mm; POD, postoperative day; *Non-parametric Wilcoxon test for paired samples. **(F)** Time course of Antithrombin (AT) activity (%). Chromogenic test; POD, postoperative day; *Non-parametric Wilcoxon test for paired samples.

The ROTEM^®^ analysis showed the following results: For the intrinsic coagulation pathways, median INTEM CFT value at ICU admission was prolonged compared to the median baseline value (*p* < 0.0001), however results remained within the institutional reference range (Figure 3A). INTEM alpha-angle dropped before ICU Admission (*p* < 0.0001) but recovered by POD 14 (*p* < 0.0001) (Figure 3B). INTEM CFT as well as INTEM alpha-angle measured before LVAD implantation had a strong, significant correlation to total blood loss (Table 4). For the extrinsic coagulation pathways the EXTEM MCF and EXTEM alpha-angle examined before LVAD implantation showed a strong and significant correlation to total blood loss (Table 4).

EXTEM alpha-angle showed a significant (*p* < 0.0001) initial drop until ICU admission and increased significantly until POD 21 (Figure 3C) whereas the EXTEM MCF values were relatively constant till the end of the fist postoperative week and then showed a significant increase till the 14th POD above the reference range (Figure 3D). EXTEM- alpha-angle as well as -MCF values examined before LVAD implantation showed a moderate correlation to total blood loss (Table 4). Patients undergoing surgical bleeding revision had reduced preoperative EXTEM alpha values compared to patients without surgical bleeding revision (74 IQR (69–74) vs. 79 IQR (78–81); *p* = 0.002), indicating a lower speed at which a solid clot forms. Additionally, EXTEM MCF-values examined before LVAD implantation were significantly reduced in patients undergoing surgical bleeding revision [63 IQR (57.7–63.7) vs. 67 IQR (65–75.5); *p* = 0.019].

Regarding the fibrin part of the clot, FIBTEM maximum clot firmness (MCF) values showed an initial significant drop until ICU admission, which was followed by a significant increase until POD 14 (*p* < 0.0001). Interestingly, FIBTEM MCF values were considerably above the institutional reference values from POD 3 onwards (Figure 3E), indicating an increase in clot firmness over time. FIBTEM MCF values examined before LVAD implantation showed a moderate correlation to total blood loss (Table 4).

### Results of Standard Laboratory Examinations

Of the standard laboratory parameters measured before LVAD implantation, fibrinogen levels (Clauss), antithrombin (AT) activity (%) and the platelet count (Table 4) correlated significantly with total blood loss. AT activity dropped below the reference range until ICU admission (*p* < 0.0001) and showed a significant increase by POD 21 (Figure 3F).

### Results of Thrombin Generation Measurements

**Figure 4:**
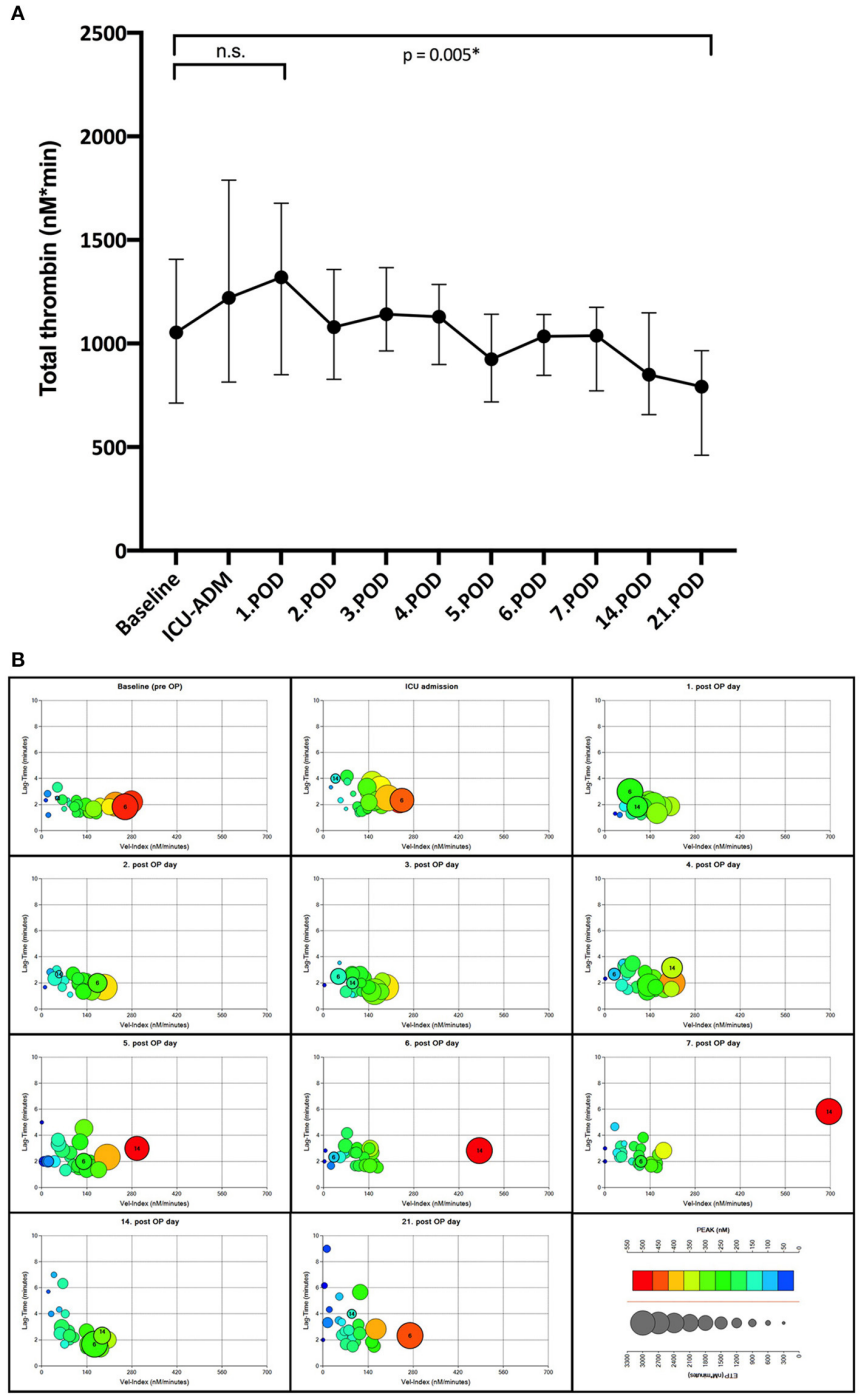
**(A)** Time course of total thrombin. Total thrombin (nM*min) as measured by Thrombinoscope^®^ (Stago Austria GmbH, Vienna); POD, postoperative day; *Non-parametric Wilcoxon test for paired samples; n.s, not significant. **(B)** Thrombin generation phenotypes in the LVAD population. Each individual in the population is defined by four thrombin parameters and their phenotype represented graphically by a positioned colored circle: *y*-axis, lag time in minutes; *x*-axis, velocity index in nM/min (rate of thrombin generation); color, peak thrombin in nM (maximum thrombin level); and size, total thrombin in nM*min (ETP or endogenous thrombin potential). Patient N°6 developed heparin-induced thrombocytopenia (HIT) at POD 16; thrombin generation of Patient N°14 was boosted approximately 10-fold by a gram*-*positive sepsis.

We observed a wide and very dynamic variation of thrombin generation profiles across the study population (Figures 4A,B). In general, total thrombin peaked on POD 1 and showed a significant drop toward the end of the observation period (Figure 4A). Total thrombin before LVAD implantation was neither associated with bleeding (Table 4) nor was it significantly associated with TRAP-induced platelet activation [Spearman's ρ = −0.01 95%CI (−0.13 to 0.1), *p* = 0.81]. The patient with the highest total thrombin level (5,728 nM.min) of our cohort (N°14, Figure 4B) developed pump thrombosis during the first week after LVAD implantation in the course of a catheter-associated sepsis with *Staphylococcus epidermidis*, suggesting that monitoring thrombin generation could be helpful for assessing the individual risk of thrombosis after LVAD implantation. Patient N°6 (Figure 4B) showed a second increase of total thrombin (2,548 nM.min) almost simultaneously with the diagnosis of a heparin-induced thrombocytopenia on POD 16.

## Discussion

In our patient cohort, bleeding occurred in 7 (28%) patients following LVAD implantation, an incidence rate comparable to that reported in previous studies. As expected (4), early bleeding was mediastinal, thoracic-pleural and from the chest wall while late bleeding is mostly a result of gastrointestinal tract hemorrhage. In our recent study describing the characteristics of early bleeding revision after LVAD implantation, we noted a “diffuse bleeding” (6) in almost 64% of the cases, pointing to clinically relevant, primary hemostatic disorders. Considering the two different types of devices implanted in this cohort, it is debatable whether the implantation technique or required circulatory support significantly affected our findings. HM devices require the creation and tunneling of a subcutaneous pocket while HVADs involve a smaller dissection for the intrapericardial implantation. A careful dissection of the pump pocket as well as preserved hemostasis is therefore deemed essential to keep bleeding events low (30). Consistently, we found that total median blood loss in HM patients was significantly higher than that in HVAD patients. Data from the Intermacs registry, which includes >25,000 patients, goes in line with our observations, showing that gastrointestinal bleeding affected 25% of patients with axial flow HMII devices and 20% of patients with centrifugal flow HVAD devices (5). However, other studies have reported no difference in the overall bleeding rates between axial and pulsatile flow devices (31).

The etiologies of postoperative bleeding are multifactorial and include thrombocytopenia and activation of fibrinolytic systems. In particular, the recognition of postoperative platelet dysfunction as an important risk factor for bleeding (32) has prompted extensive research in the field of LVAD-induced platelet dysfunction in recent years (33–35). This research discovered the importance of both acquired von Willebrand syndrome (AvWS) (33, 34) and platelet receptor alterations (35) after LVAD implantation. Furthermore, in cases of clinical deterioration the LVAD implantation cannot be delayed and a sustained impact of anti-platelet medication or of anticoagulants taken before LVAD implantation that enhances the bleeding risk is highly probable. At least a platelet function assessment before LVAD implantation could therefore assist the clinicians in decision-making about the optimal timing of surgery after cessation of antiplatelet drugs in clinically stable patients and provides a better understanding of the mechanisms of bleeding for the anesthetist while managing the urgent cases in the OR. For example, an earlier transfusion of platelets in the OR could be a consequence.

Our findings reveal a moderate to strong correlation between TRAP results, INTEM CFT, INTEM alpha-angle, EXTEM alpha and EXTEM MCF values and total blood loss, suggesting that Multiplate^®^ and ROTEM^®^ analysis could help to detect a pre-existing and clinically significant disorder of the primary hemostasis (36, 37) before LVAD implantation. It is important to note that, particularly for surgically revised patients, preoperative TRAP, ADP and ASPI test values were significantly reduced. Ranucci et al. (32) reported that both, reduced values in the ADP test and the TRAP test at baseline were significantly associated with postoperative bleeding in patients after non-LVAD cardiac surgery. A threshold of below 22 U for the ADP test and below 75 U for the TRAP test has been suggested by these authors to be associated with enhanced bleeding (32). Interestingly, the subgroup of patients with ADP test <22 U, TRAP test ≥75 U was not associated with severe bleeding in that study (32). These authors conclude that in patients with reduced ADP test values, the residual platelet reactivity to thrombin stimulation limits the risk of severe postoperative bleeding (32). This could be an explanation for our observation, that not all patients with distinctly reduced preoperative ADP test values suffer from significant postoperative bleeding.

It is worth mentioning that the main criticisms to POC monitoring are their limited accuracy and reliability and their low predictive power (10, 37). A systemic review by Corredor and coworkers concluded that the use of a combination of viscoelastic methods and platelet agonist assays achieved the greatest reduction in blood loss and blood transfusion requirements after cardiac surgery (37). Furthermore, Bolliger et al. (10) explained in a recently published article that within different test assays the evaluation of the thrombin-receptor pathway are suitable to monitor basal platelet function and could therefore be used as a reference.

We can only speculate about the mechanism of reduced TRAP-induced platelet activation before LVAD implantation. Because we did not observe a significant difference in median platelet count in patients with normal or impaired platelet response, low platelet count alone is not a plausible explanation for the reduced kinetics of clot propagation and primary hemostatic dysfunction. In patients with acute ischemic stroke, Jurk et al. (38) demonstrated a refractory status of platelets to thrombin activation due to cleavage and internalization of the thrombin receptor (PAR-1) with a missing response to thrombin suggesting an exhaustion of platelets. Heart failure is a risk factor for stroke in patients with atrial fibrillation (39) and therefore one could expect a similar mechanism in our series. Another explanation may be an altered expression of platelet receptors P-selectin, GPIbα and PECAM-1 in context of oxidative stress linked to a high bleeding risk as recently described by Klaeske et al. (35). Furthermore, most of our patients were on any anti-platelet medication or anti-coagulated with LMWH or oral anticoagulation. This could have resulted in decreased responses to agonists in the Mutiplate^®^ measurements (40) before LVAD implantation and also subsequently. Particularly, P_2_Y_12_ inhibition exerts thereby a degree of PAR-1 inhibition (41). Thrombin and ADP act synergistically in the process of platelet activation, and P_2_Y_12_ receptor inhibition partially attenuates the effect of thrombin receptors activation (32). Roka-Moiia and coworkers recently reported a very interesting mechanism of shear-mediated downregulation of GPIb and P_IIb_β_3_ on platelets associated with an evident decrease of platelet aggregatory response induced by ADP and TRAP 6 (42). At least for the reduced TRAP-induced platelet aggregation after LVAD implantation observed in our series till the 14th POD this mechanism could provide a plausible explanation.

Coagulation involves activation and aggregation of platelets, as well as deposition and maturation of fibrin. Preoperative values of ROTEM^®^ EXTEM alpha, EXTEM MCF, INTEM alpha, INTEM CFT and FIBTEM MCF were correlated with total blood loss, providing evidence of low speed of clot propagation and fibrin polymerization, as well as lower clot stabilization. The negative correlation of platelet count and fibrinogen (Clauss) to total blood loss (Table 4) signifies in the same direction.

Bleeding after LVAD implantation in our series seems therefore likely a result of primary hemostatic dysfunction characterized by poor platelet function, reduced glycoprotein IIb/IIIa and fibrinogen interaction, and/or weaker clot propagation.

It should be mentioned that median INTEM α-values were in the reference range for normal values for the total observation period, whereas FIBTEM MCF values were distinctly above normal reference values from POD 3 onwards. Hence, minimal differences in INTEM α-angle values may hint at clinically significant changes in clot propagation despite being within the “normal” range. The hyperfibrinogenemia seen after POD 3 is a reflection of a pro-coagulable state independent of a recovering platelet count. To date no data is available that addresses hyperfibrinogenemia depicted by ROTEM^®^ analysis early after LVAD implantation. Our data show a strong correlation of plasma fibrinogen values with FIBTEM MCF, reflecting the contribution of fibrinogen levels to clot firmness, while verifying a good level of agreement between fibrinogen (clauss) and ROTEM FIBTEM analysis (ρ = 0.76 95%CI 0.7–0.81; *p* < 0.0001).

Unexpectedly, we found a negative correlation of preoperative AT activity levels with estimated blood loss. AT is the most important inhibitor of coagulation proteases, such as thrombin and factor Xa (43). AT drop during cardiac surgery due to hemodilution has been described elsewhere (44). Similarly, in our cohort AT levels dropped significantly after LVAD implantation. One reason for the correlation of reduced AT activity levels with enhanced total blood loss could be that the stress of LVAD implantation coupled with the stimulation of CPB or ECMO support pushes the hemostatic system in a prothrombotic direction (45) that could result in enhanced consumption of coagulation factors and finally to enhanced bleeding. It has been proposed that AT may limit consumptive coagulopathy by suppressing thrombin generation during cardiac surgery, however this is still controversially (46). AT supplementation is indicated in patients with AT deficiency to improve heparin sensitivity but should not be used prophylactically to reduce bleeding following CPB (24).

## Limitations

Our study has some limitations. As the local ethics committee required informed consent before LVAD implantation we may have inadvertently biased our results by excluding >50% of all potential study candidates, most of them with worse clinical status (i.e., patients supported by ECMO and/or intubated before LVAD implantation). This resulted in a relatively small number of patients. Furthermore, the results of correlation analysis performed between preoperative examined coagulation parameters and the total blood loss is not a proof of a causal relationship in spite of a significant correlation. In addition, chest drain output may be deemed a poor proxy for postoperative bleeding. Because drain output was related to transfusion of blood components we considered it an adequate endpoint.

A further limitation is that the accuracy and reliability of point-of-care devices to assess functional platelet activity are generally limited in the perioperative period and the predictive values for postoperative hemorrhage and transfusion requirements have been reported to be rather low (10). Additionally, significant variability between and within these tests is one of the major disadvantages (10).

Furthermore, postoperative bleeding is of multifactorial nature. Along with the reduced preoperative platelet function other factors play an important role. The impact of residual heparin, the consumption of coagulation factors including fibrinogen, different modalities of cardio-pulmonary support, different surgical techniques and also the different LVAD designs might have influenced our findings. However, we believe that the heterogeneity of our study population is—against all skepticism—one of its strength. In our opinion it rather reflects truthfully a clinical every day scenario the anesthetists, the surgeons and critical care physicians are confronted with.

## Conclusion

Multiplate^®^ and ROTEM^®^ employed before LVAD implantation as a supplementation of standard coagulation parameters may identify LVAD candidates with platelet dysfunction and alterations of the primary hemostatic function. This could guide anesthetists and intensive care practitioners in bleeding risk stratification before LVAD implantation and in the perioperative clinical management. We deem an assessment of platelet function and ROTEM^®^ examination before LVAD implantation as valuable to assist decision-making about the best timing of surgery and to reduce the exposure of the patient to an unnecessary risk for bleeding complications. Future studies should investigate the value of POC-guided therapeutic decisions and of specific cut-off values in the assessment of platelet function and for ROTEM^®^ values that could improve the timing of LVAD implantation in efforts to improve outcomes.

## Data Availability Statement

The original contributions presented in the study are included in the article/Supplementary Material, further inquiries can be directed to the corresponding author/s.

## Ethics Statement

The studies involving human participants were reviewed and approved by Ethics Committee of the Medical University of Vienna. The patients/participants provided their written informed consent to participate in this study.

## Author Contributions

PO: writing of the article, designing the study, acquiring and analyzing data, clinical care for the patient, and editing. AF: acquiring data and clinical care for the patient. CS, MD, and AZ: editing. MB: acquiring data and editing. MM: analyzing data. DZ: clinical care for the patient. BS: corresponding author, editing, acquiring data, and clinical care for the patients.

## Conflict of Interest

DZ received funding from HeartWare and Thoratec. The remaining authors declare that the research was conducted in the absence of any commercial or financial relationships that could be construed as a potential conflict of interest.

## Publisher's Note

All claims expressed in this article are solely those of the authors and do not necessarily represent those of their affiliated organizations, or those of the publisher, the editors and the reviewers. Any product that may be evaluated in this article, or claim that may be made by its manufacturer, is not guaranteed or endorsed by the publisher.
